# Royal Jelly Supplementation Improves Endurance and Mitochondrial Biogenesis in Athletes: A Crossover Trial

**DOI:** 10.1002/fsn3.70497

**Published:** 2025-07-16

**Authors:** Yahya Pasdar, Vahid Tadibi, Ehsan Sadeghi, Farid Najafi, Mohammadreza Abbaspour, Amir Saber, Zahra Ghorbani, Shima Sharifi, Mahsa Miryan

**Affiliations:** ^1^ Department of Nutritional Sciences School of Nutritional Sciences and Food Technology, Kermanshah University of Medical Sciences Kermanshah Iran; ^2^ Exercise Metabolism and Performance Lab (EMPL), Department of Exercise Physiology, Faculty of Sport Sciences Razi University Kermanshah Iran; ^3^ Research Center for Environmental Determinacies of Health (RCEDH), School of Public Health, Kermanshah University of Medical Sciences Kermanshah Iran; ^4^ Targeted Drug Delivery Research Center, Pharmaceutical Technology Institute, Mashhad University of Medical Sciences Mashhad Iran; ^5^ Department of Clinical Biochemistry School of Medicine, Kermanshah University of Medical Sciences Kermanshah Iran

**Keywords:** antioxidants, gene expression regulation, oxidative stress, physical endurance, royal jelly

## Abstract

Royal jelly (RJ) has been shown in animal models to alleviate muscle fatigue and damage during exercise. This trial was designed to assess the effect of RJ on exercise performance, oxidative stress, and nuclear factor erythroid 2‐related factor 2 (*Nrf2*) and peroxisome proliferator‐activated receptor gamma coactivator 1‐alpha (*PGC‐1α*) expression in endurance‐trained male athletes. In this randomized, crossover trial, 18 eligible participants were randomly assigned to receive RJ (1000 mg/day) in one period and placebo in another period for 2 weeks, with a 2‐week washout. The exhaustive endurance‐running test was conducted to assess time to exhaustion (TTE), perceived exertion, arousal levels, and affective response. Blood samples were collected to assess gene expression, malondialdehyde (MDA), total antioxidant capacity (TAC), and total oxidant status (TOS). The carryover and sequence effects were not significant for all outcomes (*p*‐value > 0.05). The mean changes from baseline were statistically significant for TTE (7.32; 95% CI: 4.61–10.02; *p*‐value: 0.001), post‐exercise TOS (−18.56; 95% CI: −31.61 to −5.51; *p*‐value: 0.008), and PGC‐1α expression (2.23; 95% CI: 0.49–3.98; *p*‐value: 0.015) with RJ, and TTE (2.69; 95% CI: 0.80–4.59; *p*‐value: 0.008) with placebo. The treatment differences in TTE and PGC‐1α expression relative to placebo were 4.63 min (95% CI: 1.78–7.48; *p*‐value: 0.002) and 2.12 (95% CI: 0.16–4.09; *p*‐value: 0.035), respectively. No significant effects were observed in *Nrf2* expression, perceived exertion, arousal levels, affective response, heart rate, and other oxidative stress markers. RJ supplementation improved endurance capacity and *PGC‐1α* expression but had no significant effect on oxidative stress and *Nrf2* expression in endurance‐trained athletes, suggesting selective ergogenic effects. Further studies are needed to evaluate its efficacy across different athlete populations.

## Graphical abstract

This clinical trial showed that royal jelly supplementation significantly improved athletic performance by increasing the time to exhaustion and peroxisome proliferator‐activated receptor gamma coactivator 1‐alpha (*PGC‐1α*) gene expression during the exhaustive endurance‐running test. However, its supplementation had no significant effects on nuclear factor erythroid 2‐related factor 2 (*Nrf2*) gene expression, malondialdehyde (MDA), total antioxidant capacity (TAC), total oxidant status (TOS), heart rate, rate of perceived exertion (RPE), arousal, and pleasure to exercise [Up Arrow (Increase); Double‐sided Arrow (Without Change)].

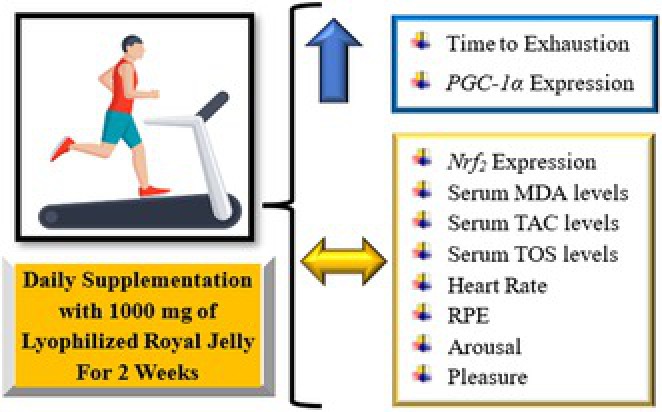

## 1 Introduction

Mitochondria mainly contribute to supplying the energy requirements for muscle contraction through the oxidative phosphorylation pathway. Mitochondrial dysfunctions have been implicated in sarcopenia, diabetes, and cardiovascular diseases (Ferri et al. 2020; Ren et al. 2010). Muscular mitochondrial content also directly influences physical performance by increasing oxidative capacity, endurance, muscle strength, and force production (Carter et al. 2015). Although regular exercise is the main strategy for optimizing mitochondrial biogenesis, some functional foods and nutraceuticals are emerging as complementary approaches. These not only increase mitochondrial biogenesis but also neutralize reactive oxygen species (ROS) overproduced during exercise (Broome et al. 2024; Martín‐Rodríguez et al. 2024). Moreover, studies in recent years have shown that phytochemicals can mitigate oxidative stress and inflammation in critically ill patients (Malekahmadi et al. 2021; Pahlavani et al. 2022), improve exercise performance in athletes (Putera et al. 2023), and reduce cardiometabolic risk factors in the general population (Jandari et al. 2020).

Royal jelly (RJ), a nutritious secretion of honeybees ( *Apis mellifera* ), is becoming increasingly popular for managing chronic illnesses and enhancing human health. Fresh RJ consists of about 50%–70% moisture, 9%–18% proteins, 7%–18% carbohydrates, and 3%–8% lipids, along with different minerals, vitamins, and phytochemicals (Kanelis et al. 2024). It has been marketed as a functional food and dietary supplement specific to improving both healthspan and lifespan. The antioxidant, anti‐inflammatory, anti‐tumor, and anti‐aging activities of RJ have been established in in vitro and in vivo models (Kumar et al. 2024; Oršolić and Jazvinšćak Jembrek 2024). Meta‐analytic evidence from clinical trials has shown that RJ can be a safe and effective dietary supplement for controlling glycemia in diabetic patients (Bahari, Taheri, Rashidmayvan, Hezaveh, et al. 2023; Bahari, Taheri, Rashidmayvan, Jamshidi, et al. 2023). Moreover, the latest findings revealed that the administration of RJ can decrease the risk of cardiovascular diseases (Bahari, Taheri, Rashidmayvan, Hezaveh, et al. 2023; Bahari, Taheri, Rashidmayvan, Jamshidi, et al. 2023) and alleviate gastrointestinal diseases (El‐Seedi et al. 2024). In neurodegenerative models, RJ improved glial and neuronal cell survival and restored cognitive impairments (Ali and Kunugi 2020).

RJ has shown potential in preventing age‐related declines in muscle mass, strength, and motor function in male mice through the regulation of both regenerative and degenerative genes (Okumura et al. 2018). RJ has been shown to increase the time to exhaustion (TTE), reduce serum lactate concentrations, and maintain muscle glycogen during endurance exercise in mice (Kamakura et al. 2001). RJ administration also increases mitochondrial biogenesis during endurance exercise in mice by activating the AMP‐activated protein kinase (AMPK) (Takahashi et al. 2018). This activation upregulates the peroxisome proliferator‐activated receptor gamma coactivator 1‐alpha (*PGC‐1α*), which acts as the master regulator of oxidative metabolism and mitochondrial biogenesis (Jäger et al. 2007). Furthermore, the beneficial effects of RJ administration against excessive ROS production in skeletal muscles following exhaustive exercise have been evaluated in in vivo models (Bakır and Bakır 2023; Magholi et al. 2022). In addition to scavenging ROS, RJ has been shown in rats to upregulate the expression of the nuclear factor erythroid 2‐related factor 2 (*Nrf2*) gene, a key regulator of the enzymatic antioxidant genes (Parlak et al. 2023).

Recent research has shown that sports supplements containing RJ enhance high‐intensity interval exercise (HIIE) performance in swimmers by influencing biomarkers of oxidative stress and muscle damage (Ovchinnikov, Paoli, et al. 2022). However, there are few clinical studies on the impact of RJ on exercise performance. In this context, RJ supplementation has been shown to attenuate the decline of muscle strength in elderly individuals and improve the aerobic capacity of sedentary individuals (Meng et al. 2017; Taşdoğan et al. 2020). These results do not necessarily translate to a competitive advantage for athletes over that achieved by regular training alone, as the impact of RJ supplementation might be greater in non‐athletic or elderly individuals. To address these knowledge gaps, we designed a crossover trial to evaluate the impact of RJ on athletic performance, oxidant and antioxidant biomarkers, and the expression of *Nrf2* and *PGC‐1α* genes in endurance‐trained male athletes. We hypothesized that RJ would improve TTE and *PGC‐1α* expression without altering oxidative stress markers.

## 2 Materials and Methods

### 2.1 Study Design and Participants

This randomized, double‐blind, placebo‐controlled, two‐way crossover trial assessed the efficacy of RJ on athletic performance, oxidative and antioxidant capacity, and the expression of *Nrf2* and *PGC‐1α* in endurance‐trained male athletes. This clinical trial was carried out at the Sports Laboratory of Razi University in Kermanshah, Iran. Participants were recruited through advertisements on social media, announcements at local sports facilities and universities, and collaboration with sports organizations in Kermanshah city.

All participants completed an incremental treadmill running test (H/P Cosmos Sports & Medical Pulsar 3p 4.0 treadmill, Germany) during the screening phase to select eligible participants and evaluate maximal aerobic speed (MAS) (Machado et al. 2013). The procedure began with a warm‐up stage, including walking at a speed of 6 km/h for 3 min. After the warm‐up, the treadmill started at 8 km/h and increased by 1 km/h every 3 min. The incline was consistently maintained at 1°. Participants continued the test until they could no longer proceed, even with verbal encouragement. The MAS was calculated from the final speed and duration obtained during the test, along with participants reaching 90% of their maximum heart rate (220 minus age) and achieving a rate of perceived exertion (RPE) of 19. Participants who sustained the incremental treadmill running test for at least 18 min were eligible for inclusion in the trial.

Other inclusion criteria were as follows: male gender; individuals aged young to middle adulthood; and participation in regular endurance activity at least three times per week for the past 6 months. The exclusion criteria included a history of allergy to bee products or RJ; metabolic, respiratory, cardiovascular, and musculoskeletal diseases; a history of physical injuries during exercise; use of anti‐inflammatory drugs and dietary supplements (such as vitamin E, vitamin C, selenium, caffeine, and RJ) within the last 3 months; and adherence to specific nutritional programs. Participants who had poor compliance (defined as 80% or less) with the assigned treatment or withdrew their consent after randomization were excluded from the trial.

The trial was conducted in accordance with the provisions of the Declaration of Helsinki, and written informed consent was obtained from all participants. The trial protocol was approved by the Research Ethics Committee of Kermanshah University of Medical Sciences (ID: IR.KUMS.REC.1402.419) and has been registered at the Iranian Registry of Clinical Trials (ID: IRCT20231209060310N1). This trial follows the CONSORT guidelines for crossover designs, data analyses, and reporting. The detailed protocol for the trial has been published previously (Miryan et al. 2025).

### 2.2 Randomization and Blinding

**FIGURE 1:**
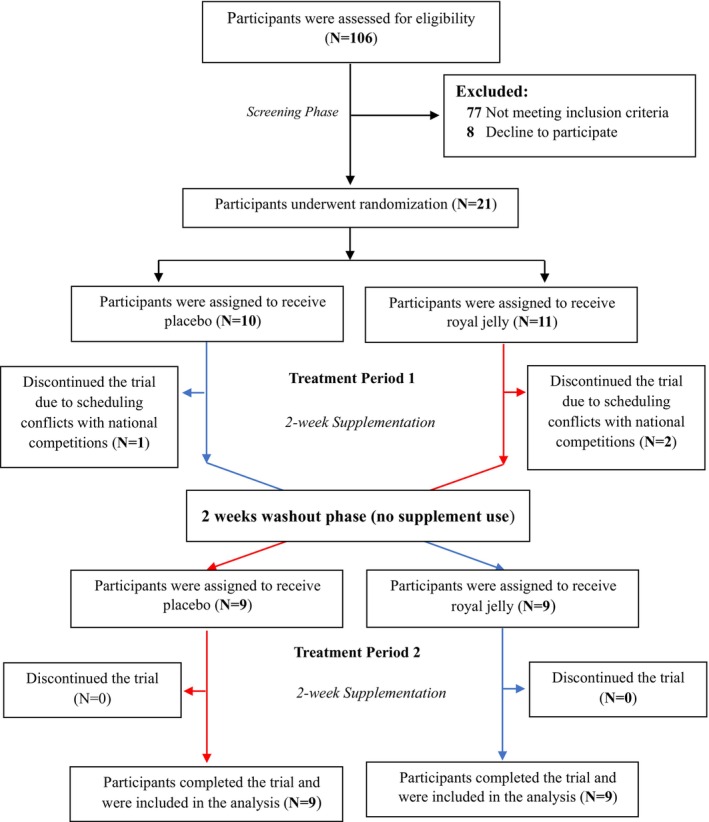
Screening, randomization, treatment, and follow‐up.

Participants were randomly assigned in a 1:1 ratio to take RJ capsules during treatment period 1 for 2 weeks and then placebo capsules in treatment period 2 for 2 weeks (RJ–Placebo sequence), or to receive placebo capsules in treatment period 1 for 2 weeks and then RJ capsules in treatment period 2 for 2 weeks (Placebo–RJ sequence). The trial flowchart is shown in Figure 1. Randomization was conducted using computer‐generated random numbers prepared by an independent statistician. The randomization sequences were written on cards and then kept in sealed, opaque, sequentially numbered envelopes. These envelopes were opened sequentially for eligible participants after obtaining consent. Participants and researchers who were involved in enrollment, assessments, and data analyses were unaware of the randomization sequences and treatments until the study was completed and the data had been analyzed.

### 2.3 Intervention

Participants were instructed to consume RJ capsules for 2 weeks in one treatment period and placebo capsules for 2 weeks in another treatment period, taken before breakfast and dinner. Each RJ capsule contained 500 mg of lyophilized RJ, while the placebo capsule contained whole corn flour. RJ and placebo capsules were identical in packaging, color, odor, size, weight, and appearance. Participants received daily text messages to remind them to take capsules and follow the study protocol. At the end of each treatment period, compliance rates were calculated by counting the remaining capsules in each participant's supplement bottle.

In the present clinical trial, we administered a daily dose of 1000 mg of lyophilized RJ for 2 weeks, as previous research has shown the beneficial effects of RJ at this dose and duration on the aerobic capacity of sedentary individuals with no reported adverse events (Taşdoğan et al. 2020). A 2‐week washout period was implemented following treatment period 1. While the half‐life of RJ has not been established in the scientific literature, many studies involving dietary supplements in athletes typically employ a 2‐week washout phase, which is generally considered sufficient to eliminate any carryover effects (Keong et al. 2006; Zhou et al. 2005). More information is available in the Trials Journal (Miryan et al. 2025).

### 2.4 Assessment of Athletic Performance

Eligible participants underwent an exhaustive endurance‐running test to assess exercise performance according to the TTE at the beginning and end of each treatment period. The exhaustive endurance‐running test was performed on the H/P Cosmos Sports & Medical Pulsar 3p 4.0 treadmill at a constant speed set at 80% of the MAS achieved during an incremental treadmill running test. The MAS was calculated using the following formula (Machado et al. 2013):

$$\mathrm{MAS}=V_{\text{complete}}+\left(\mathrm{inc}\times t/T\right)$$

where *V*_complete_: Running speed at the last stage that the participant completed fully in the incremental treadmill test; inc, speed increment from one stage to the next (1 km/h); the number of seconds spent in the incomplete stage; *T*, the total number of seconds required to complete a full stage.

During the exhaustive endurance‐running test, researchers regularly assessed participants and stopped the treadmill if any issues arose. After walking at 6 km/h for 3 min (warm‐up), participants began running on a treadmill at a constant speed set at 80% of their MAS until they reached exhaustion. If a participant reached 90% of the maximum heart rate and an RPE of 19 but was unable to continue the activity, the duration was noted as TTE in minutes (Machado et al. 2013).

We also measured psychophysiological feedback to exercise, including perceived exertion, affective response, and arousal levels, during the exhaustive endurance running test using validated visual analog scales. Perceived exertion was assessed using the RPE scale, ranging from 6 (minimum exertion) to 20 (maximum exertion). The affective response was assessed using the feeling scale (FS), which ranged from −5 (maximum displeasure) to 5 (maximum happiness). Perceived arousal was measured using the felt arousal scale (FAS) with scores between 1 (minimum) and 6 (maximum). Participants reported RPE, FS, and FAS scores every 3 min and at the end of the test. For all three measures, mean values were calculated to determine overall perceived exertion, affective response, and arousal scores (Astorino et al. 2012; Bastos et al. 2023). Participants were asked to maintain a consistent diet regimen and avoid exercise for 3 days before the exhaustive endurance‐running test to minimize differences in muscle glycogen and fatigue.

### 2.5 Measurement of Oxidative Stress Biomarkers

Blood samples were drawn from the cubital vein before and after the exhaustive endurance running test at the beginning and end of each trial period. The samples were immediately centrifuged at 3000 rpm for 10 min at 4°C, and then the separated serum was stored at −20°C until analysis. Malondialdehyde (MDA), total antioxidant capacity (TAC), and total oxidant status (TOS) were analyzed using the colorimetric technique with commercial kits (Kiazist, Iran). Serum MDA levels were measured based on the reaction between MDA and thiobarbituric acid. Serum TAC levels were measured according to the ability of antioxidants to convert cupric cations (Cu^2+^) to cuprous cations (Cu^+^). Serum TOS levels were measured based on the potencies of oxidants to convert ferrous cations (Fe^2+^) to ferric cations (Fe^3+^).

### 2.6 Nrf2 and PGC‐1α mRNA Expression

#### 2.6.1 Peripheral Blood Mononuclear Cells (PBMCs) Isolation

The blood samples were collected from participants using sterile tubes containing ethylene diamine tetraacetic acid (EDTA) before each exhaustive endurance‐running test. Then, blood samples were mixed with phosphate‐buffered saline (PBS), layered on top of Ficoll solution (Inno‐train, Germany), and subjected to centrifugation at 600*g* for 20 min at 25°C. PBMCs were isolated from the second layer (Jia et al. 2018; Li et al. 2022). Total RNA was extracted from PBMCs. TRIzol and chloroform were added to each sample tube. The upper aqueous phase was transferred to a new tube following mixing and centrifugation. Isopropanol was added, and the samples were centrifuged to precipitate RNA. The RNA pellet was washed with 70% ethanol dissolved in diethyl pyrocarbonate‐treated water (DEPC‐treated water).

#### 2.6.2 RNA Quality

RNA concentration and purity were analyzed using a NanoDrop spectrophotometer (Thermo Fisher Scientific, USA). RNA samples with a 260/280 ratio ranging from 1.8 to 2.1 were considered suitable, demonstrating high RNA purity without detectable protein or phenol contamination, and were therefore used in this study.

#### 2.6.3 Primer Design

*Nrf2, PGC‐1α*, and beta‐actin (*ACTB*) gene sequences were ascertained on NCBI GenBank (www.ncbi.nlm.nih.gov/genbank), and specific primer sequences were designed using OLIGO 7 software and were validated via BLAST analysis on the NCBI website for their validity.

The primer sequences used in this study were as follows:

- *PGC‐1α* forward (5′‐ACCTACCGTTATACCTGTGA‐3′)
- *PGC‐1α* reverse (5′‐TCCACAAAAGTACAGCTCAAA‐3′)
- *Nrf2* forward (5′‐CACATCCAGTCAGAAACCAGTG‐3′)
- *Nrf2* reverse (5′‐CTACAAACGGGAATGTCTGCG‐3′)
- *ACTB* forward (5′‐ATGACTTAGTTGCGTTACACC‐3′)
- *ACTB* reverse (5′‐AAACAAATAAAGCCATGCCAA‐3′)

#### 2.6.4 Complementary DNA (cDNA) Synthesis

RNA was reverse‐transcribed into cDNA using the Easy cDNA Synthesis Kit, ParsTous, Iran. Based on the manufacturer's instructions, a 20 μL volume was used for the reaction, which consisted of 1 μL of total RNA template, 10 μL of buffer mix, 2 μL of enzyme mix, and 7 μL of DEPC‐treated water. The thermal cycling protocol included 10 min of initial incubation at 25°C, 10 min of annealing of primers and cDNA synthesis at 47°C, and 5 min of enzyme inactivation at 85°C. At the end of the procedure, samples were cooled down to 4°C and stored at −20°C until needed for real‐time polymerase chain reaction (RT‐PCR) analysis.

#### 2.6.5 RT‐PCR

For evaluating the relative expression of mRNA levels of *Nrf2* and *PGC‐1α* genes in this study, we used mRNA expression levels on an RT‐PCR system by utilizing the 2^−ΔΔCt^ method (Livak and Schmittgen 2001) and were normalized to ACTB, serving as the housekeeping gene (Li et al. 2022).

*ACTB* was selected as the housekeeping gene for this study based on prior evidence showing its consistent expression in humans, especially in PBMCs (Hazarika et al. 2023). To confirm its stability under our experimental conditions, we carried out a preliminary RT‐PCR analysis, which revealed uniform expression levels across all treatment conditions. These results support the reliability of *ACTB* as a reference gene in our setup and align with current recommendations emphasizing the need to validate reference genes within the specific context of each experiment.

### 2.7 Dietary Intake Assessment

Participants were instructed to complete a food record for 3 days before the exhaustive endurance‐running test. Dietary intakes were converted from portion sizes into grams using the Iranian household measurements and then transformed into daily energy and nutrients using Nutritionist IV software (First Databank, Hearst Corp, San Bruno, CA, USA).

### 2.8 Preparation of Supplements

In this clinical trial, fresh RJ was harvested from beehives (*Apis mellifera*) and immediately stored at −20°C to preserve its nutritional properties. It was sourced from Negin Shahd Sepahan (Isfahan, Iran). In the next stage, the moisture content of the RJ was completely removed using the freeze‐drying method, transforming it into a uniform powder suitable for capsulation. In this process, the RJ is initially frozen, and then under vacuum conditions, its water is directly converted from ice to vapor without damaging bioactive compounds in the RJ. Fresh RJ contained about 60% moisture. Finally, the lyophilized RJ was automatically capsulated and packaged. To prepare the placebo capsules, whole corn flour was used to closely match the color of lyophilized RJ. RJ and placebo capsules were manufactured by the School of Pharmacy at Mashhad University of Medical Sciences, Mashhad, Iran.

The 10‐hydroxy‐2‐decenoic acid (10‐HDA) has been recognized as the main bioactive compound in RJ (Guo et al. 2021). The level of 10‐HDA in RJ is considered a hallmark of RJ quality (Muñoz et al. 2011). We measured 10‐HDA and other components of this lyophilized RJ at the reference laboratory, Hortash, in Isfahan, Iran. These results showed that lyophilized RJ consisted of 40.55% carbohydrate, 40.50% protein, 15.08% total fat (7.9% 10‐HDA and 7.18% other fats), 2.78% ash, and 1.09% moisture. Whole corn flour, being biologically inert and having negligible physiological effects, is frequently employed as a placebo in studies (Whitney et al. 2019). The placebo contained 76% carbohydrate, 8.5% protein, 4% fat, 1.2% ash, and 10.3% moisture.

### 2.9 Statistical Analysis

We estimated that a sample size of 15 participants would provide the trial with more than 80% power to detect an effect size (*δ*) of 1.4 nmol/mL for serum MDA levels with a standard deviation (*σ*) of 1.38, using a two‐tailed alpha level of 0.05 (Miryan et al. 2025). Considering a dropout probability of 20% due to the longitudinal design, a total of 18 participants were initially enrolled. The sample size was calculated using the following formula (Sakpal 2010):

$$N=\frac{2\times \sigma^{2}\times {\left(\frac{Z_{\alpha }}{2\;}+Z_{\beta }\;\right)}^{2}}{\delta^{2}}$$

Continuous variables were examined for normality of distribution using the Shapiro–Wilk test. Participant characteristics at baseline were compared using the independent‐sample t‐test (normal distribution) and the Mann–Whitney U test (non‐normal distribution). Within‐group comparisons (changes in outcomes from baseline) were conducted using the paired‐sample t‐test (normal distribution) and the Wilcoxon signed‐rank test (non‐normal distribution). The carryover effect—whether RJ supplementation in treatment period 1 influenced the results obtained in treatment period 2—was assessed by checking the assumptions of the Linear Mixed Model (LMM) in Stata Software (version 17.0). When it was not statistically significant for each outcome, again, LMM was run to assess treatment, period, and sequence effects using IBM SPSS Statistics Software (Version 22). This model included treatments (RJ vs. Placebo), periods (first vs. second), sequences (RJ–Placebo vs. Placebo–RJ), and covariates (energy intake, age, and baseline values) as fixed factors, and participants as a random factor. Data are reported as means ± standard error (SE) or means with 95% confidence intervals. All tests were two‐tailed, and statistical significance was defined as *p*‐value < 0.05.

## 3 Results

In this trial, 21 eligible participants were assigned to take RJ capsules (*n* = 11) or placebo capsules (*n* = 10) during the treatment period 1. Among these participants, 1 from the placebo treatment and 2 from the RJ treatment discontinued the trial and did not complete the exhaustive endurance running test at the end of treatment period 1 due to scheduling conflicts with national competitions, which made physical attendance for testing impossible. A total of 18 participants—9 in the placebo treatment and 9 in the RJ treatment—completed the trial protocol in both treatment periods and were included in the data analyses. The mean compliance rate was 91.3% for RJ capsules and 94.6% for placebo capsules. No adverse events were reported during the trial. The trial's flowchart is shown in Figure 1.

All participants had a higher education level, with a mean ± SE age of 26.83 ± 2.30 years and a BMI of 22.97 ± 0.54 kg/m^2^. The baseline demographics, anthropometrics, and biochemical measurements are shown in Table 1. Demographic and anthropometric characteristics at baseline were balanced between the RJ–Placebo and Placebo–RJ sequences. The TOS levels at baseline were significantly higher in the RJ–Placebo sequence compared to the Placebo–RJ sequence, while the TAC/TOS ratio was lower. No significant differences were observed in serum TAC or MDA between sequences (*p*‐value > 0.05). The analysis of dietary intake obtained from 3 days before each exhaustive endurance‐running test is shown in Table 2. The intake of energy and nutrients did not differ significantly between the first and second running tests in both treatments (*p*‐value > 0.05).

**TABLE 1:** Baseline demographic, anthropometric, and clinical characteristics of participants by treatment sequence and by total.

Variables	Total	Treatment sequence		*p*
		Placebo to RJ	RJ to placebo	
Number, *n*	18	9	9	—
Age, years	26.83 ± 2.30	26.83 ± 3.75	24.33 ± 2.61	0.290
Weight, kg	75.70 ± 2.07	73.23 ± 3.02	78.17 ± 2.75	0.244
Height, cm	181.52 ± 1.86	179.66 ± 2.77	183.38 ± 2.48	0.332
BMI, kg/m^2^	22.97 ± 0.54	22.66 ± 0.67	23.29 ± 0.89	0.583
WC, cm	79.75 ± 1.59	81.66 ± 2.01	77.83 ± 2.42	0.241
HC, cm	96.75 ± 1.25	96.16 ± 1.54	97.33 ± 2.06	0.657
Waist‐to‐hip ratio	0.82 ± 0.01	0.84 ± 0.01	0.80 ± 0.02	0.256
Body fat mass, kg	13.07 ± 1.14	13.11 ± 1.52	12.94 ± 1.84	0.944
Soft lean mass, kg	55.86 ± 3.11	55.88 ± 2.85	55.83 ± 5.74	0.993
Total body water, kg	45.22 ± 1.35	43.32 ± 2.15	47.13 ± 1.49	0.165
SBP, mmHg^a^	127.05 ± 2.41	124.33 ± 4.03	129.77 ± 2.58	0.272
DBP, mmHg^a^	70.22 ± 2.09	69.33 ± 2.69	71.11 ± 3.36	0.685
Sport duration, h/day	1.88 ± 0.11	1.83 ± 0.16	1.94 ± 0.15	0.631
History of sport, years	11.44 ± 2.06	11.94 ± 2.30	10.94 ± 3.56	0.437
BEE, Kcal/day	1673.01 ± 41.94	1610.33 ± 71.72	1735.66 ± 36.77	0.140
TAC, nmol/mL^a^	2140.30 ± 85.68	2223.70 ± 144.75	2056.90 ± 92.53	0.346
TOS, nmol/mL^a^	76.23 ± 4.86	64.65 ± 4.33	87.81 ± 6.95	0.012
MDA, nmol/mL^a^	17.39 ± 0.75	17.47 ± 1.08	17.31 ± 1.13	0.919
TAC/TOS ratio^a^	29.66 ± 1.76	34.75 ± 1.69	24.75 ± 1.96	0.001
Incremental running test, min	20.03 ± 0.58	20.26 ± 0.98	19.81 ± 0.68	0.716

*Note:* Plus‐minus values are means ± standard errors. *p*‐values were calculated with the use of an independent‐sample *t*‐test.

Abbreviations: BEE, basal energy expenditure; BMI, body mass index; bpm, beats per minute; DBP, diastolic blood pressure; HC, hip circumference; MDA, malondialdehyde; RJ, royal jelly; SBP, systolic blood pressure; TAC, total antioxidant capacity; TOS, total oxidant status; WC, waist circumference.

^a^ Data were measured before the exhaustive endurance running test.

**TABLE 2:** Comparisons of dietary intake obtained 3 days before each exhaustive endurance‐running test in the RJ condition (*N* = 18) and the placebo condition (*N* = 18).

Variables	Conditions	Exhaustive endurance‐running test		*p*
		First	Second	
Energy, Kcal/day	RJ	2221 ± 136.07	2186 ± 94.99	0.785
	Placebo	2141 ± 134.06	2015 ± 118.19	0.288
Protein, g/day	RJ	94.11 ± 11.18	94.20 ± 7.33	0.991
	Placebo	88.49 ± 7.85	91.40 ± 8.51	0.667
Carbohydrate, g/day	RJ	281.58 ± 19.52	263.20 ± 16.62	0.492
	Placebo	258.22 ± 19.78	235.80 ± 16.37	0.232
Fat, g/day	RJ	79.88 ± 6.18	84.05 ± 5.09	0.216
	Placebo	83.82 ± 5.38	78.56 ± 4.57	0.398
Saturated fat, g/day	RJ	19.77 ± 1.71	21.26 ± 2.29	0.598
	Placebo	19.82 ± 1.76	18.67 ± 1.01	0.494
Monounsaturated fat, g/day	RJ	22.16 ± 2.20	24.68 ± 1.77	0.434
	Placebo	24.90 ± 1.80	22.87 ± 1.52	0.367
Fiber, g/day	RJ	19.70 ± 1.99	19.68 ± 2.09	0.994
	Placebo	18.97 ± 1.74	17.48 ± 1.96	0.409
Calcium, mg/day	RJ	477.93 ± 42.88	597.71 ± 60.09	0.140
	Placebo	597.29 ± 75.78	568.28 ± 73.73	0.694
Magnesium, mg/day	RJ	233.93 ± 24.73	233.80 ± 19.78	0.994
	Placebo	226.73 ± 18.56	222.17 ± 19.59	0.820
Potassium, mg/day	RJ	2331 ± 204.47	2284 ± 147.13	0.853
	Placebo	2371 ± 169.41	2196 ± 169.68	0.314
Sodium, mg/day	RJ	2601 ± 125.77	2692 ± 175.63	0.673
	Placebo	2756 ± 230.75	2628 ± 174.07	0.548
Zinc, mg/day	RJ	9.45 ± 2.11	8.82 ± 1.16	0.659
	Placebo	9.42 ± 1.32	9.82 ± 1.40	0.669
Selenium, mg/day	RJ	0.11 ± 0.06	0.14 ± 0.08	0.756
	Placebo	0.11 ± 0.06	0.12 ± 0.06	0.288
Vitamin C, mg/day	RJ	71.76 ± 23.25	61.29 ± 13.36	0.506
	Placebo	76.25 ± 14.43	61.48 ± 16.81	0.102
Vitamin E, mg/day	RJ	12.01 ± 2.58	12.13 ± 2.67	0.965
	Placebo	12.28 ± 2.79	9.70 ± 2.02	0.336

*Note:* Plus‐minus values are means ± standard errors. *p*‐values were calculated with the use of a paired sample *t*‐test.

Table 3 shows the mean changes in oxidative stress biomarkers from baseline and the adjusted treatment differences (RJ minus Placebo). The mean changes from baseline in pre‐exercise levels of TAC, TOS, MDA, and TAC/TOS ratio were not significant in both treatments (*p*‐value > 0.05). In the RJ treatment, post‐exercise levels of TOS significantly reduced from baseline (−18.56 ± 6.18; *p*‐value: 0.008), while the TAC/TOS ratio increased (5.20 ± 2.00; *p*‐value: 0.019). LMM showed no significant carryover, treatment, period, and sequence effects for oxidative stress biomarkers (*p*‐value > 0.05).

**TABLE 3:** Mixed model comparisons between the RJ and placebo conditions on oxidative stress biomarkers throughout the trial.

Variables	RJ (*N* = 18)		Placebo (*N* = 18)		Difference in change^b^ (RJ–placebo)	*p*
	At the end of intervention	Change from baseline^a^	At the end of intervention	Change from baseline^a^		
Pre‐exercise test						
TAC, nmol/mL						
Mean ± SE	2192.1 ± 79.55	93.21 ± 85.35	2184.68 ± 80.07	−66.72 ± 95.29	52.68 ± 116.13	0.654
(95% CI)		(−86.86–273.28)		(−267.77–134.31)	(−185.8–290.2)	
TOS, nmol/mL						
Mean ± SE	71.34 ± 4.03	−9.80 ± 6.45	75.52 ± 3.89	7.91 ± 5.32	−2.31 ± 6.39	0.720
(95% CI)		(−23.43–3.82)		(−3.31–19.15)	(−15.38–10.76)	
MDA, nmol/mL						
Mean ± SE	17.10 ± 0.54	−0.22 ± 1.05	17.89 ± 0.77	0.06 ± 0.89	−0.75 ± 1.00	0.456
(95% CI)		(−2.43–1.99)		(−1.83–1.94)	(−2.80–1.28)	
TAC/TOS ratio						
Mean ± SE	31.50 ± 2.33	3.93 ± 1.96	30.46 ± 2.11	−3.37 ± 2.39	2.57 ± 2.70	0.350
(95% CI)		(−0.21–8.08)		(−8.41–1.67)	(−2.97–8.11)	
Post‐exercise test						
TAC, nmol/mL						
Mean ± SE	2147.6 ± 75.44	−60.49 ± 94.62	2211.2 ± 63.84	−123.45 ± 76.96	−38.06 ± 94.12	0.689
(95% CI)		(−260.14–139.15)		(−285.84–38.93)	(−230.5–154.4)	
TOS, nmol/mL						
Mean ± SE	65.85 ± 4.39	−18.56 ± 6.18	70.16 ± 2.66	−5.75 ± 4.29	−6.89 ± 4.20	0.112
(95% CI)		(−31.61 to −5.51)*		(−14.81–3.31)	(−15.49–1.70)	
MDA, nmol/mL						
Mean ± SE	18.23 ± 0.66	1.32 ± 1.02	16.56 ± 0.72	−0.04 ± 0.95	1.76 ± 0.94	0.071
(95% CI)		(−0.84–3.48)		(−2.07–1.97)	(−0.16–3.69)	
TAC/TOS ratio						
Mean ± SE	33.96 ± 1.64	5.20 ± 2.00	32.04 ± 1.21	−0.54 ± 2.17	2.65 ± 2.12	0.222
(95% CI)		(0.97–9.43)*		(−5.13–4.04)	(−1.69–7.00)	

Abbreviations: CI, confidence intervals; MDA, malondialdehyde; RJ, royal jelly; SE, standard error; TAC, total antioxidant capacity; TOS, total oxidant status.

^a^ Mean changes were calculated with the use of a paired *t*‐test.

^b^ Mean differences were calculated by a linear mixed model with participants as a random effect, randomization sequence, period, and treatment as fixed effects, and energy intake, age, and baseline values as covariates.

* *p*‐value < 0.05.

**FIGURE 2:**
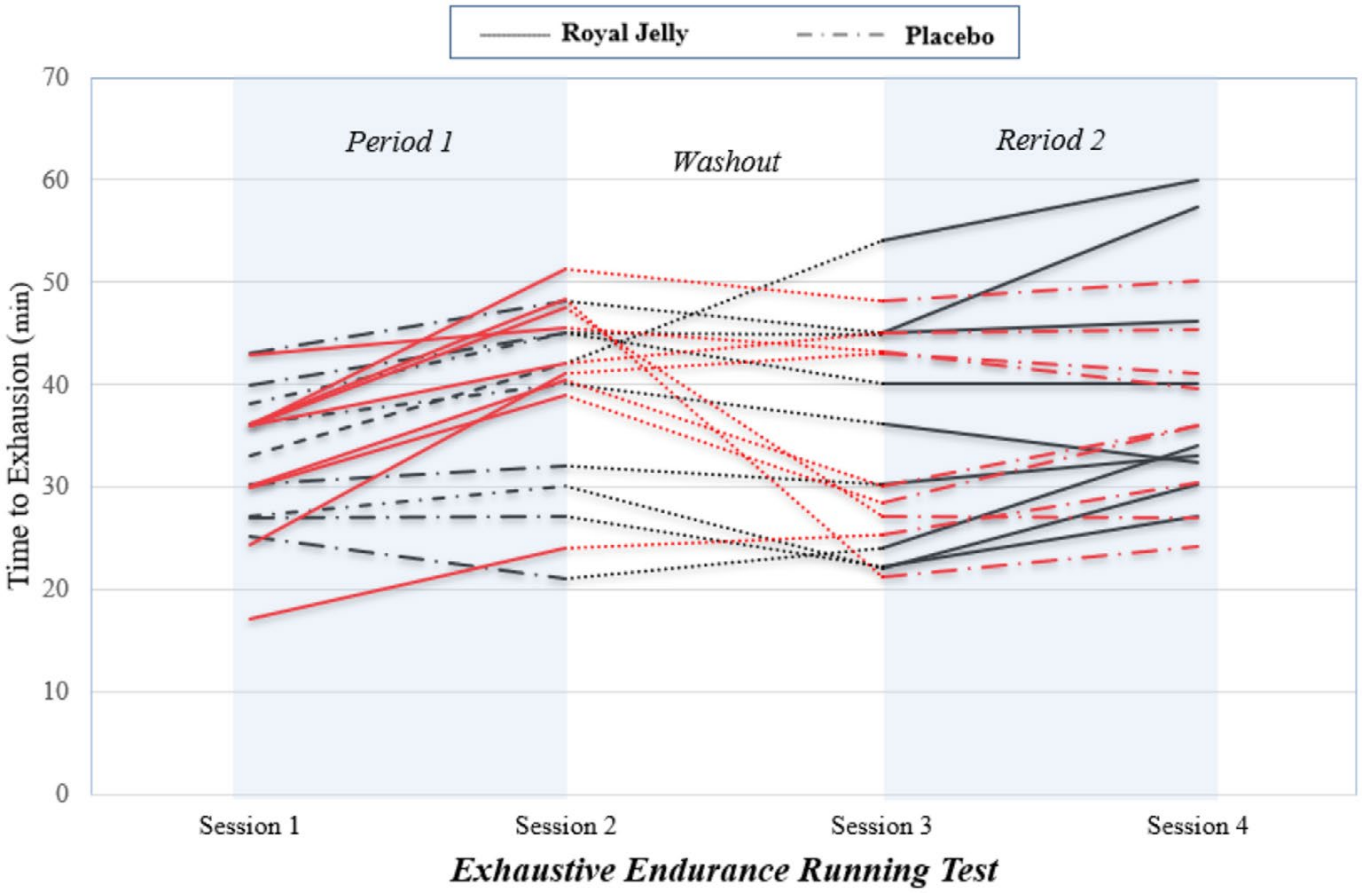
Time to exhaustion throughout the trial. Red lines represent participants in the royal jelly to placebo sequence, while black lines represent those in the placebo to the royal jelly sequence.

The mean changes in athletic performance from baseline are shown in Table 4. The mean TTE significantly increased from baseline in the RJ treatment (7.32 ± 1.28; *p*‐value: 0.001) and the placebo treatment (2.69 ± 0.89; *p*‐value: 0.008). The mean changes from baseline in RPE, FS, and FAS scores were not significant in both treatments. The carryover and sequence effects were not significant for all these variables (*p*‐value > 0.05). The treatment effect was significant for TTE. RJ supplementation significantly increased TTE compared to placebo, with an adjusted mean difference of 4.63 (95% CI: 1.78–7.48; *p*‐value: 0.002). Figure 2 depicts the trends of TTE according to participants, sequences, periods, and treatments throughout the trial.

**TABLE 4:** Mixed model comparisons between RJ and placebo on athletic performance during the exhaustive endurance‐running test.

Variables	RJ (*N* = 18)		Placebo (*N* = 18)		Difference in change^b^ (RJ–placebo)	*p*
	At the end of intervention	Change from baseline^a^	At the end of intervention	Change from baseline^a^		
Time to exhaustion, min						
Mean ± SE	41.12 ± 2.33	7.32 ± 1.28	36.74 ± 2.05	2.69 ± 0.89	4.63 ± 1.39	0.002
(95% CI)		(4.61–10.02)*		(0.80–4.59)*	(1.78–7.48)	—
Perceived exertion						
Mean ± SE	12.36 ± 1.80	0.27 ± 0.46	12.55 ± 1.29	−0.25 ± 0.37	0.12 ± 0.52	0.824
(95% CI)		(−0.71–1.25)		(−1.02–0.51)	(−0.96–1.20)	—
Affective response						
Mean ± SE	2.01 ± 0.27	−0.63 ± 0.36	2.16 ± 0.24	−0.07 ± 0.17	−0.37 ± 0.30	0.241
(95% CI)		(−1.39–0.13)		(−0.43–0.30)	(−0.99–0.26)	—
Arousal levels						
Mean ± SE	3.81 ± 0.20	−0.28 ± 0.17	3.96 ± 0.17	−0.04 ± 0.18	−0.20 ± 0.22	0.385
(95% CI)		(−0.64–0.07)		(−0.43–0.35)	(−0.67–0.27)	—
Heart rate, bpm						
Mean ± SE	170.18 ± 2.07	1.31 ± 1.70	171.72 ± 2.30	4.69 ± 2.58	−2.24 ± 2.82	0.433
(95% CI)		(−2.27–4.91)		(−0.76–10.14)	(−8.03–3.53)	—

Abbreviations: bpm, beats per minute; CI, confidence intervals; RJ, royal jelly; SE, standard error.

^a^ Mean changes were calculated with the use of a paired *t*‐test.

^b^ Mean differences were calculated by a linear mixed model with participants as a random effect, randomization sequence, period, and treatment as fixed effects, and energy intake, age, and baseline values as covariates.

* *p*‐value < 0.05.

**FIGURE 3:**
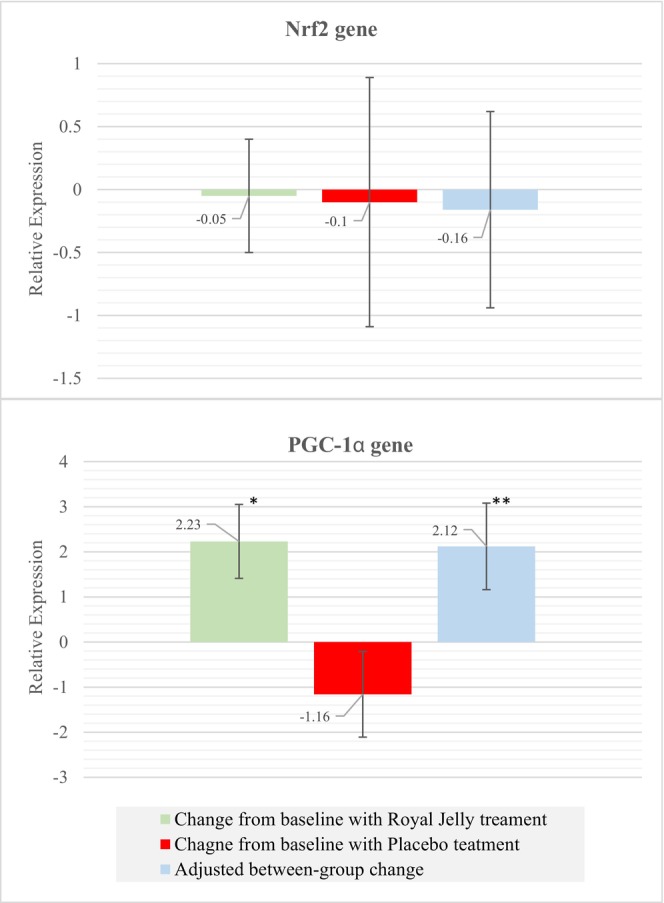
Change from baseline and adjusted between‐group change in the relative expression of *Nrf2* and *PGC‐1α* genes. Data are shown as means and standard error bars. *Significant changes from baseline. **Significant differences in changes (RJ vs. Placebo).

The mean changes in the *Nrf2* and *PGC‐1α* expression are indicated in Figure 3. No significant changes from baseline in the *Nrf2* expression were seen in both conditions (*p*‐value > 0.05). The relative expression of the *PGC‐1α* mRNA significantly increased from baseline in the RJ treatment (2.23 ± 0.82; *p*‐value: 0.015), while it was unchanged in the placebo treatment (−1.16 ± 0.95; *p*‐value: 0.457). The carryover, period, and sequence effects were not significant for these genes (*p*‐value > 0.05). The treatment effect was significant for the expression of *PGC‐1α* mRNA. RJ supplementation significantly increased *PGC‐1α* gene expression compared to placebo, with an adjusted mean difference of 2.12 (95% CI: 0.16–4.09; *p*‐value: 0.035). More detailed information is presented in Table 5.

**TABLE 5:** Mixed model comparisons between RJ and placebo on gene expression levels during the exhaustive endurance‐running test.

Variables	RJ (*N* = 18)		Placebo (*N* = 18)		Difference in change^b^ (RJ–placebo)	*p*
	At the end of intervention	Change from baseline^a^	At the end of intervention	Change from baseline^a^		
*Nrf2*						
Mean ± SE	2.09 ± 0.49	−0.05 ± 0.45	2.33 ± 0.61	−0.10 ± 0.99	−0.16 ± 0.78	0.831
(95% CI)		(−1.00–0.91)		(−2.18–2.00)	(−1.77–1.43)	—
*PGC‐1α*						
Mean ± SE	3.68 ± 0.71	2.23 ± 0.82	2.43 ± 0.70	−1.16 ± 0.95	2.12 ± 0.96	0.035
(95% CI)		(0.49–3.98)*		(−3.16–0.84)	(0.16–4.09)	—

Abbreviations: bpm, beats per minute; CI, confidence intervals; *Nrf2*, nuclear factor erythroid 2‐related factor 2; *PGC‐1α*, peroxisome proliferator‐activated receptor gamma coactivator 1‐alpha; RJ, royal jelly; SE, standard error.

^a^ Mean changes were calculated with the use of a paired *t*‐test.

^b^ Mean differences were calculated by a linear mixed model with participants as a random effect, randomization sequence, period, and treatment as fixed effects, and energy intake, age, and baseline values as covariates.

* *p*‐value < 0.05.

## 4 Discussion

In this randomized, double‐blind, placebo‐controlled, crossover clinical trial involving endurance‐trained athletes, RJ supplementation significantly improved athletic performance by increasing TTE during the exhaustive endurance‐running test. A similar benefit of RJ was seen in the relative expression of *PGC‐1α*. However, its supplementation had no significant effects on antioxidant status, oxidative biomarkers, and the relative expression of the *Nrf*_*2*_ gene. To the best of our knowledge, this is the first clinical trial to specifically evaluate the impact of RJ supplementation on athletic performance, antioxidant status, oxidative biomarkers, and the expression of *PGC‐1α* and *Nrf2* genes in endurance‐trained athletes.

Our clinical trial revealed that daily supplementation with 1000 mg of lyophilized RJ (equivalent to 2500 mg of fresh RJ containing 40% solids) resulted in a significant mean increase of 4.63 min in TTE during the exhaustive endurance‐running test. Notably, this ergogenic benefit occurred without negatively affecting heart rate, perceived exertion, affective response, or arousal levels. These psychophysiological feedbacks typically deteriorate in response to an increase in the duration of high‐intensity exercise (Larumbe‐Zabala et al. 2020). The maintenance of affective response and arousal levels implies that RJ supplementation might reduce psychological strain under high‐intensity exercise, while stable heart rate and perceived exertion indicate enhanced metabolic efficiency (Astorino et al. 2012; Bastos et al. 2023). Consistent with our findings, a randomized clinical trial showed that muscle strength was increased after supplementation of protease‐treated RJ (1200 mg/day) in elderly individuals (Meng et al. 2017). Furthermore, another randomized clinical trial indicated that daily supplementation with 1000 mg of lyophilized RJ for 15 days increased the aerobic power output in sedentary males (Taşdoğan et al. 2020). RJ may also work with other nutrients to improve exercise performance under high‐intensity conditions. A randomized clinical trial showed that supplementation of RJ (400 mg) with coenzyme Q10 (60 mg) for 10 days significantly reduced serum lactate levels and the time to complete HIIE in runners (Ovchinnikov, Deryugina, and Paoli 2022). This c‐supplementation also improved the time to complete HIIE in swimmers and reduced serum creatine kinase levels (Ovchinnikov, Paoli, et al. 2022). While no significant changes were observed in markers of oxidative stress, this enhancement in performance indicates that RJ might affect alternative physiological mechanisms, such as energy metabolism.

RJ and 10‐HDA have been found to activate AMPK and increase glucose uptake in the skeletal muscles of mice (Takahashi et al. 2018; Takikawa et al. 2013). AMPK serves as a vital energy sensor within cells, and its activation is linked to numerous positive physiological outcomes, especially regarding exercise performance (Garcia and Shaw 2017). Indeed, AMPK upregulates the PGC‐1α protein as the master regulator of mitochondrial biogenesis and oxidative phosphorylation (Jäger et al. 2007). Our trial also indicated that RJ supplementation increased the *PGC‐1α* gene expression in endurance‐trained athletes. Muscular mitochondrial content directly influences exercise performance by increasing the capacity for oxidative phosphorylation, endurance, and force production (Carter et al. 2015). Therefore, the potential mechanism underlying the effects of RJ on exercise performance may be related to the upregulation of the *PGC‐1α* gene expression. This subsequently increases mitochondrial biogenesis and aerobic capacity and improves energy metabolism, allowing athletes to sustain higher intensities of exercise for longer periods.

**FIGURE 4:**
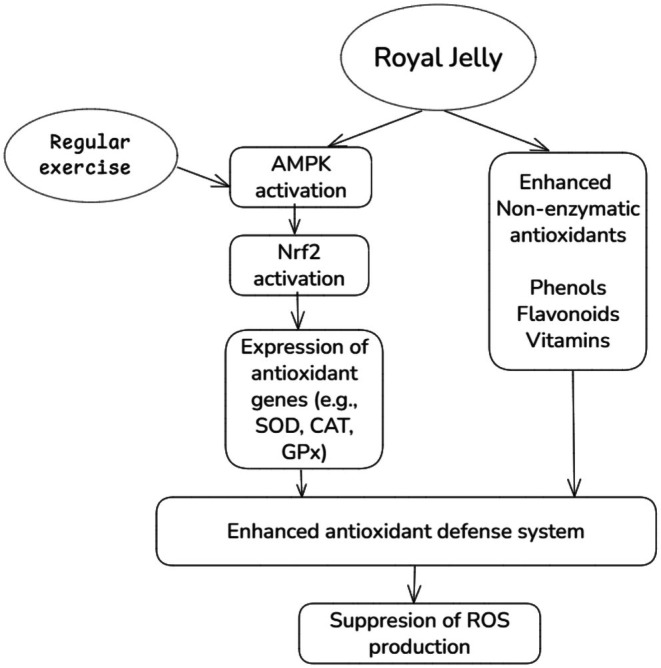
Potential mechanisms of royal jelly (RJ) on oxidative stress. RJ may reduce oxidative stress during endurance exercise through activation of the AMPK pathway and enhancement of exogenous antioxidants. However, regular exercise may upregulate the AMPK pathway and minimize the impact of short‐term administration of RJ on oxidative stress status in trained athletes. The low ATP/AMP ratio is a key signal for AMPK activation during exercise. AMPK, 5′ adenosine monophosphate‐activated protein kinase; CAT, catalase; GPx, glutathione peroxidase; Nrf2, nuclear factor erythroid 2‐related factor 2; ROS, reactive oxygen species; SOD, superoxide dismutase.

Our clinical trial indicated that RJ supplementation did not significantly affect serum levels of oxidative biomarkers and antioxidant status, including TAC, TOS, MDA, or the TAC/TOS ratio. Oxidative stress acts as a stimulator of Nrf2, which upregulates various enzymatic antioxidant and detoxification genes (Tonelli et al. 2018). In line with unchanged oxidative stress status following RJ supplementation, no significant change was observed in the expression of the *Nrf2* gene in our trial. Despite the well‐known antioxidant activities of RJ in in vitro models, previous clinical trials have produced challenging results. A clinical trial showed that RJ administration at a dosage of 1000 mg for 2 months did not significantly alter serum TAC and MDA levels in patients with type 2 diabetes mellitus (T2DM) (Shidfar et al. 2015). However, a clinical trial in T2DM patients found that daily supplementation with of RJ (1000 mg) for 2 months increased serum superoxide dismutase (SOD) and glutathione peroxidase (GPx) and reduced serum MDA concentrations (Pourmoradian et al. 2014). Another clinical trial showed that supplementation with 666 mg/day of lyophilized RJ (containing 4% 10‐HDA) for 8 weeks significantly increased serum TAC concentrations and down‐regulated the SOD gene while having no significant effect on the expression of catalase, glutathione reductase (GR), and GPx genes among overweight adults (Petelin et al. 2019). These inconsistencies are perplexing and may be attributed to differences in study populations, intervention durations, and supplement dosages. In addition, the quality of RJ supplements may explain some inconsistent findings between trials that had similar RJ dosages, intervention durations, and study populations (Pourmoradian et al. 2014; Shidfar et al. 2015). Previous research indicated that the harvest time of RJ significantly impacts its antioxidant activity, and harvesting within 24 h leads to maximum antioxidant activity (Liu et al. 2008). With knowledge of this issue, we harvested fresh RJ from beehives within 24 h and immediately stored it at −20°C. Furthermore, we included trained athletes who engaged in regular endurance activity at least three times per week for the past 6 months in this trial. A growing body of evidence highlights the role of regular exercise and training in antioxidant defense systems. Recent meta‐analytic evidence from clinical trials has shown that regular training (three sessions per week for more than 4 months) can enhance the activity of SOD and GPX enzymes, improve TAC, and reduce lipid peroxidation levels (Xie et al. 2025). These changes may be mediated by exercise‐induced activation of the Nrf2 pathway, which leads to upregulation of endogenous antioxidants (Powers et al. 2024). As a result, trained athletes might possess a better antioxidant capacity to scavenge oxidants during exercise. Also, exercise‐induced ROS does not have completely detrimental roles. Physiological concentrations of ROS are essential for optimal force production in skeletal muscles during exercise (Powers and Jackson 2008). Antioxidant‐mediated depletion of ROS within muscles reduces force production and impairs adaptations to exercise, such as muscle mitochondrial biogenesis (Higgins et al. 2020). Regular exercise can lead to adaptations in skeletal muscles to overcome increased oxidative stress by upregulating antioxidant enzymes and improving mitochondrial biogenesis (Gomes et al. 2020; Xie et al. 2025). Therefore, in addition to short‐term supplementation of RJ in our trial, these coexisting adaptations in athletes compared to other populations may minimize the impact of RJ on overall antioxidant capacity and oxidative stress status. The role of regular exercise and RJ supplementation in oxidative stress status is summarized in Figure 4.

This trial had several strengths, including a double‐blind, randomized, placebo‐controlled, crossover design; well‐defined inclusion criteria; a high completion rate (RJ: 90% & Placebo: 94.7%); a high compliance rate (RJ: 91.3% & Placebo: 94.6%); no carryover effects for all outcomes due to an adequate washout period; collection of dietary intake data; and adjustments for potential confounding factors. An additional strength of this clinical trial is the investigation of the potential biological mechanisms involved in the beneficial effects of RJ on athletic performance. A limitation of this study was the relatively small sample size; however, the crossover design increases the precision of the treatment effect estimate and helps control for inter‐individual variability (e.g., genetics, age, and coexisting conditions) rather than the parallel design with a larger sample size. In addition, the participants we enrolled were endurance‐trained athletes, so the generalizability of the findings to other athletes may be limited. Also, our findings point to a possible involvement of the AMPK‐PGC‐1α pathway activation and muscular mitochondrial biogenesis in the effect of RJ on the endurance performance of male athletes. However, this study did not directly evaluate them due to the ethical constraints regarding performing muscle biopsies. Future trials should determine the impacts of RJ administration on different athlete populations and confirm these potential pathways.

## 5 Conclusion

This randomized, double‐blind, placebo‐controlled, crossover clinical trial indicated that daily supplementation with 1000 mg of lyophilized RJ for 2 weeks significantly increased TTE and improved endurance and *PGC‐1α* gene expression but did not alter oxidative stress status or *Nrf2* gene expression, suggesting selective ergogenic effects in endurance‐trained athletes. Future trials should determine the impacts of RJ administration on different athlete populations and the underlying mechanisms.

## Author Contributions

**Yahya Pasdar:** conceptualization (equal), investigation (equal), methodology (equal), project administration (equal), supervision (equal), writing – original draft (equal). **Vahid Tadibi:** investigation (equal), methodology (equal), project administration (equal), writing – original draft (equal). **Ehsan Sadeghi:** conceptualization (equal), investigation (equal), methodology (equal), project administration (equal). **Farid Najafi:** formal analysis (equal), investigation (equal), methodology (equal), project administration (equal), writing – review and editing (equal). **Mohammadreza Abbaspour:** conceptualization (equal), investigation (equal), methodology (equal), resources (equal). **Amir Saber:** data curation (equal), formal analysis (equal), investigation (equal), methodology (equal), validation (equal). **Zahra Ghorbani:** conceptualization (equal), data curation (equal), investigation (equal), methodology (equal), resources (equal). **Shima Sharifi:** data curation (equal), investigation (equal), methodology (equal). **Mahsa Miryan:** conceptualization (equal), formal analysis (equal), funding acquisition (equal), investigation (equal), methodology (equal), project administration (equal), software (equal), validation (equal), visualization (equal), writing – original draft (equal), writing – review and editing (equal).

## Ethics Statement

The trial was conducted in accordance with the provisions of the Declaration of Helsinki, and written informed consent was obtained from all participants. The trial protocol was approved by the Research Ethics Committee of Kermanshah University of Medical Sciences (ID: IR.KUMS.REC.1402.419) and has been registered at the Iranian Registry of Clinical Trials (ID: IRCT20231209060310N1).

## Consent

Consent is available for publication.

## Conflicts of Interest

The authors declare no conflicts of interest.

## Data Availability

The data that support the findings of this study are available on request from the corresponding author. The data are not publicly available due to privacy or ethical restrictions.
